# Comparative Study on the Anticancer Drug Potential of a Lectin Purified from *Aloe Vera* and Aloe-Emodin

**DOI:** 10.31557/APJCP.2020.21.1.99

**Published:** 2020

**Authors:** Nuriye Akev, Eda Candoken, Serap Erdem Kuruca

**Affiliations:** 1 *Department of Biochemistry, Faculty of Pharmacy, *; 2 *Department of Physiology, Istanbul Faculty of Medicine, Istanbul University, Istanbul, Turkey. *

**Keywords:** Aloe, emodin, cytotoxicity, gastric adenocarcinoma, lectin

## Abstract

**Background:**

The effect of Aloctin, a lectin purified from *Aloe vera* leaves, and aloe emodin an anthraquinone glycoside purified from the leaves of the same plant, on several cancer cell lines was investigated.

**Methods:**

Aloctin was isolated from *A. vera *leaf skin by ammonium sulphate precipitation and CNBr-Sepharose 4B-ovalbumin affinity chromatography. Specific new ligands for Aloctin were detected as fetuin and avidin by hemagglutination inhibition tests. The cytotoxic effect of Aloctin and aloe emodin on various human cancer cell lines was tested using MTT assay. Imatinib was tested as standard positive control. The mechanism underlying was tested by the Annexin V-FITC/PI test, with flow cytometry.

**Results:**

The most sensitive cells to Aloctin and aloe emodin treatment, were identified as AGS human gastric adenocarcinoma cells. The effect was concentration dependent. It was shown that this effect does not occur by apoptosis or necrosis. In Aloctin-imatinib combinations studies, Aloctin significantly increased the cytotoxic effect of imatinib in a dose-dependent manner. It is expected that the results of this study will reveal important findings for the future use of *A. vera *lectin as well as aloe emodin in cancer research and contribution to lectin biochemistry.

## Introduction

As cancer continues to be one of the devastating illnesses of our century, plant kingdom with its diversity, is also researched in regard of cytotoxic and anticancer agents. Chemotherapeutic drugs of plant origin are promising strategy for cancer therapy because they might be generally harmless or less toxic than synthetic chemotherapeutic agents on normal cells.

The multiple biological effects of the “wonder plant” *Aloe vera* (L.) Burm. f. (Xanthorraceae, formerly Aloaceae and Liliaceae) were proved and reviewed by scientific research (Akev et al., 2015). Several in vivo studies were conducted from 1980’s to date with *A. vera* leaf extracts regarding their antitumour effects (Winters et al., 1981; Gribel and Pashinskiĭ, 1986; Akev et al., 2007a). 

Aloe emodin (AE) is an anthraquinone glycoside purified from *A. vera*. In addition to its well established laxative effect, AE have been reported to exhibit antiviral, antimicrobial, hepatoprotective and anticancer properties. Considerable attention has been given recently to the possibility of utilizing AE as a chemotherapeutic drug for the treatment of various types of cancers (Yordanova and Koprinarova, 2014).

Lectins are proteins or glycoproteins that bind to specific sugar residues on cell surfaces (Sharon and Lis, 1972). Lectins play multiple roles in inter- and intra-cellular signaling, cell transformation and cell adhesion (Seyrek and Bildik, 2001). It is reported that cell surface carbohydrates take place in normal and malignant transformations. Changes in cell surface glycoproteins were reported during malignant transformation (Diani, 2010). Bovin and Gabius (1995) proposed that tumours could be better recognized by some glycoprotein-binding lectin markers. Lectin-targeted chemotherapy as well as lectin binding studies for normal and tumour cells are thus promising strategies for the future (Gorelik et al., 2001). Nevertheless direct antitumour and cytotoxic effect of some lectins is also reported in literature (Hajtó et al 2005; Akev et al., 2007b; Faheina-Martins et al 2012).

The present study was undertaken in order to determine and to compare the cytotoxic effects of Aloctin, purified by CNBr-Sepharose 4B-ovalbumin affinity chromatography, and AE on several cancer cell lines.

## Materials and Methods

*Plant Material*

Specimens of *A. vera* (L.) Burm. f. were collected from Kale (Demre) in Antalya (May 1993) and cultivated in the Greenhouse of Istanbul University Alfred Heilbronn Botanical Garden. A voucher specimen was deposited in the Herbarium of the Faculty of Pharmacy, ISTE No. 65118. The fresh leaves of this cultivated plant were used in the study.

*Preparation of A. vera leaf extract*

Freshly chopped *A. vera* leaves, were washed carefully with water and dried with filter paper (Whatmann 41) to remove dust and foreign materials. The leaves were put vertically in a becher for one night in order to discard the brown latex rich in anthraquinones. Then the leaves were longitudinally split in two, the gel was separated by scraping with a spoon, the leaf skins were cut into little pieces and homogenized with phosphate buffered saline (PBS) in a Waring blendor. The homogenate was filtered through cloth and then filtrate was centrifuged at -10°C, 10.000 rpm for 30 min. The clear supernatant was lyophylized and considered to be “*A. vera* leaf skin crude extract”.

*Purification of A. vera lectin*

The lectin was precipitated by adding 50% ammonium sulphate to the *A. vera* leaf skin crude extract solution. The precipitate was dissolved in a minimum amount of PBS, dialyzed against PBS and centrifuged. The clear supernatant was named “50% ammonium sulphate cut”. 

*Affinity chromatography on CNBR-activated Sepharose 4B-ovalbumin*

50% ammonium sulphate cut was applied to affinity chromatography on a column of CNBR-activated Sepharose 4B-ovalbumin. The protein peak of the eluate showing hemagglutination was collected, concentrated, lyophilized and the purity was assessed by polyacrylamide gel electrophoresis as described previously (Ozsoy et al., 2012).

*Hemagglutination inhibition tests*

They are hapten inhibition tests performed in order to investigate the inhibition of lectin-induced hemagglutination and thus find the proper ligand of the lectin. The hemagglutination inhibition tests by various carbohydrates were performed in a manner analogous to the hemagglutination test (Wang et al. 1995; Akev and Can 1999). The carbohydrates used were D-maltose, D(+)-raffinose, mellibiose, D-mannose, N-acetyl-D-galactosamine and chitin. The glycoproteins used were: fetuin, avidin, musin and inulin.

*Preparation of test materials and reference drugs*

Aloe emodin (AE) (1,8-dihydroxy-3-[hydroxymethyl]-anthraquinone) was purchased from Sigma (St Louis, MO, cat no. A7687). AE (20 mmol/L) stock solutions were prepared in DMSO, aliquoted and stored in the dark at −20°C till use, diluted with medium. Imatinib (IM) was a kind gift from Istanbul University, Istanbul Faculty of Medicine, Department of Physiology. 

*Cell lines and cell culture*

AGS human gastric adenocarcinoma, HCT116 human colon cancer, HEP3B human hepatoma, HL60 human acute promyelocytic leukemia, K562 human chronic myelogenous leukemia and Saos-2 human osteosarcoma cell lines were a courtesy of Prof. Dr. Serap Erdem Kuruca, Istanbul University, Istanbul Faculty of Medicine, Department of Physiology. The cells were cultured in DMEM (Dulbecco’s Modified Eagle’s medium; Sigma-Aldrich) supplemented with 10 % fetal bovine serum (FBS; Capricorn FBS-12A), 100 units/mL penicillin and 100 μg/mL of streptomycin in a humidified incubator containing 5 % CO_2_ at 37°C. In order to reach the sufficient cell number for tests, cells were passaged after reaching 80% monolayer confluency. Cells were sub-cultured every 2 or 3 days.

*Trypan Blue exclusion assay*

The total number of viable cells was determined at each time point by the trypan blue exclusion test (Strober 2001). Exactly 10 μL of cell suspensions was stained with an equal volume of trypan blue (0.4 % in 10 mM phosphate buffer saline) for 1 min. Then the numbers of viable cells were counted with Neubauer Chamber by light microscopy. Cells that retained a blue color were considered as dead cells. 

*MTT colorimetric assay*

The MTT colorimetric assay developed by Mosmann 1983 with modification was used to screen for cytotoxic activity. For this purpose 96-well plate was used and the assay was done in a total volume of 100 μL. Briefly, 10 μL/well of varying concentrations of AVG and AE, (50 - 250 μg/mL; 10 – 80 µM respectively) were added and subsequently the cells (90 µL/well; 105 cells/mL culture medium) were seeded to treate for 72 h. After the aspiration of supernatant (50 µL/well), incubation with MTT solution (10 μL of 5 mg/mL PBS) at 37°C for 3 h, cells were lyzed with 100 μL dimethyl sulfoxide (DMSO). The yellow MTT dye was reduced by succinic dehydrogenase in the mitochondria of viable cells to purple formazan crystals. Absorbance was measured at 570 nm using a microplate reader. 

To account for absorbance of samples at 570 nm, during each MTT experiment, separate wells were set where samples were diluted in culture medium without cells. The average absorbance readings from wells containing samples in culture medium were subtracted from the readings of treated cells. To calculate viability index, absorbance readings from DMSO treated control wells were set at 100% and the relative absorbance was calculated as a percentage of the control.

The results were generated from three independent experiments; all experiments were performed in triplicate. Cytotoxic index was expressed as a percentage relative to the untreated control cells.

The cytotoxic concentrations of extracts that provides 50% inhibition of cell growth (IC_50_) were calculated from dose-response curve. The cytotoxic effect of *A. vera* extracts and controls were evaluated by comparing the IC_50_ values of cell lines.

*Flow cytometry analysis*

Fluorescein (FITC) Annexin V-/ Propidium iodide (PI) double labeling was performed with the Annexin V-FITC apoptosis detection kit (Millipore) to detect the apoptosis of cells. For this purpose 6-well plate was used and the assay was done in a total volume of 2 mL. The three groups (two untreated control cells group: to apply and unapplied Annexin V-FITC/PI for one test group) of cells (1800 µL/well; 10^5^ cells/mL culture medium) were seeded in 6-well plates in a final concentration of IC_50_ (200 μL/well) of *A. vera* gel extract and AE. After culture at 37°C with 5% CO_2_ for 72 h, the cells were harvested by trypsinization. Prior to trypsinization, floating or loose cells were harvested by gentle manual rocking of the culture dishes and transferring the culture medium containing the cells into centrifuge tubes. Trypsinized and loose cells were then combined and pelleted by centrifuging at 2000 rpm for 10 min. The pellets were resuspended and washed with PBS, then resuspended in 100 µL of Annexin Binding Buffer (4X) and stained with 3 µL Annexin V-FITC, 2 µL PI. The cell suspension was incubated for 45 min at room temperature in the dark. The cell suspension was then immediately analyzed by flow cytometry. Cell Quest software was used to analyze 10^4^ cells. The apoptotic cells were determined with a FACS Calibur flow cytometer (BD Biosciences) and analyzed with CELLQUEST software (BD Biosciences).

*Statistical analysis*

The results were statistically analyzed using the independent Student’s t-test. Data were represented as means ± standard deviation (S.D.) and at least in triplicate. Results were considered significant with P < 0.05 (*), P < 0.01 (**) ve P < 0.001 (***).

## Results

*Affinity chromatography purification of A. vera lectin (Aloctin)*

**Figure 1:**
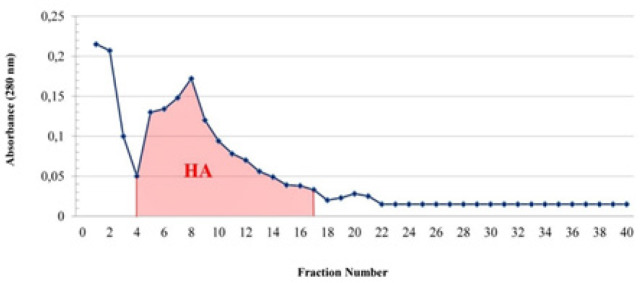
Elution Profile of *Aloe vera* Lectin (Aloctin) Purification. Affinity chromatography of 50% ammonium sulphate precipitate of *Aloe vera* leaf skin extract using CNBr- Sepharose 4B- ovalbumin. HA, Hemagglutination activity; Column size, 25x0.5 cm; Flow rate, 4 ml/min. :Absorbance (280 nm); HA, Fractions showing hemagglutination activity

The affinity chromatography elution profile of Aloctin purification is shown in Figure 1. The lectin was eluted as a single peak. The results of Aloctin purification steps are shown in Table 1.

None of the carbohydrates tested showed inhibition of hemagglutination. Only N-acetyl D-galactosamine showed weak inhibition as found previously (Akev and Can 1999). Among the glycoproteins tested fetuin and avidin significantly inhibited hemagglutination activity of Aloctin and their minimum inhibitory concentrations are 0.156 mg/ml and 0.078 mg/ml, thus are potential ligands for further studies with Aloctin. Results of hemagglutination inhibition test are shown in Table 2.

*Cytotoxicity of Aloctin against cancer cell lines.*

The cytotoxic concentrations of Aloctin that provides 50% inhibition of cell growth (IC_50_) are shown in Table 3. The lowest IC_50_ value shows the better cytotoxic effect. Thus we can range the cells sensitive to Aloctin as AGS > Saos-2 > HEP3B > K562 > HL-60 > HCT116. 

**Figure 2:**
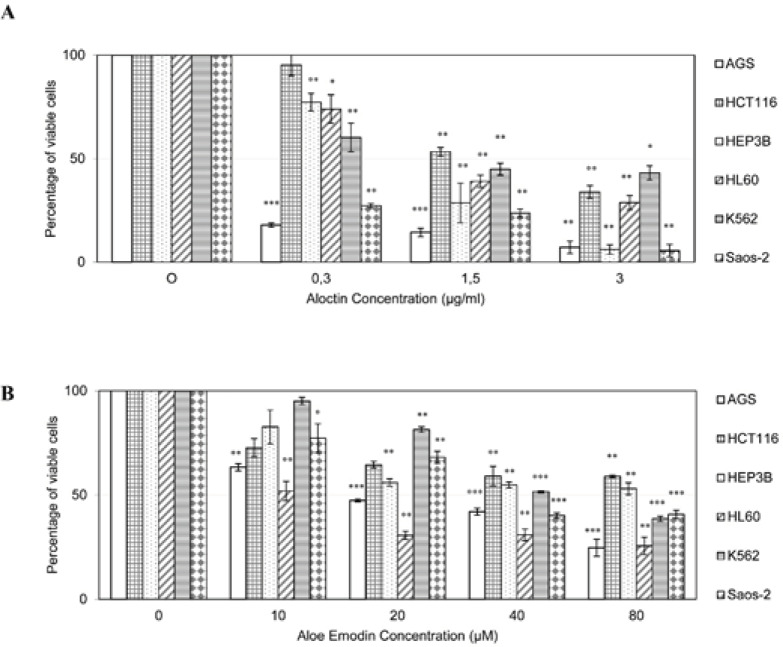
Cell Viability (%) Level Measured by MTT Assay after 72 Hours Treatment with Different Concentration of Aloctin (A) and Aloe Emodin (B) on Cancer Cells. 400, 800 and 1000 μg/ml concentrations showed significant differences, compared to control group. AGS human gastric adenocarcinoma cells, HCT116 human colon cancer cells, HEP3B human hepatoma cells, HL60 human acute promyelocytic leukemia cells, K562 human chronic myelogenous leukemia cells, Saos-2 human osteosarcoma cells. *P < 0.05; **P < 0.01; ***P < 0.001

**Figure 3:**
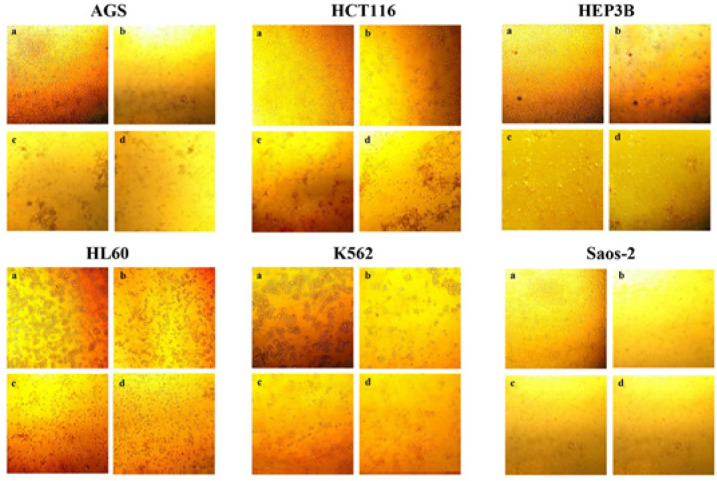
Inverted Microscope Images of Cell Confluency in Different Doses of Aloctin at 72 Hours. The cells were treated with different doses of Aloctin for 72 hours. Cell viability was significantly reduced, in comparison with control (a), by the dose in 0.3 (b), 1.5 (c) and 3 (d) μg/ml concentrations. (Magnification ×100, scale bar=1 μm)

The cytotoxic effect of Aloctin on the cells as percentage of viable cells, is shown in Figure 2A. The best effect was shown on AGS cells human gastric adenocarcinoma cells whereas HCT116 human colon cancer cells were resistant to Aloctin treatment. The cytotoxic effect was concentration dependent as shown in Figure 3.

*Cytotoxicity of standard reference drugs against cancer cell lines*

The cytotoxic concentrations of Aloe emodin (AE) and Imatinib (IM) that provides 50% inhibition of cell growth (IC_50_) are shown in Table 3. The best effect of IM was seen on K562 cells whereas the effect of AE could be ranged as AGS > HL60 > Saos-2 > K562 > HCT116 > HEP3B cells.

*Cytotoxicity of aloe emodin against cancer cell lines*

The cytotoxic effect of AE on the cells as percentage of viable cells, is shown in Figure 2B. Significant cytotoxic effect was shown with treatment of AGS cells with AE after 10 µM concentration (P < 0.01). On HCT116 cells significant cytotoxicity was observed only at higher doses of 60-150 µM (P < 0.01). On HEP3B cells significant cytotoxicity was observed after doses of 20 µM (P < 0.01). Cytotoxic activity of AE on HL60 cells was significant between 20-80 µM (P < 0.01). AE showed significant cytotoxicity on K562 cells at 20 µM (P < 0.01) and between 40 – 80 µM (P < 0.001) concentrations. AE showed significant cytotoxicity towards Saos-2 cells at lower doses of 10 µM (P < 0.05), and also at higher doses of 20 µM (P < 0.01), 40 µM (P < 0.001), 60µM (P < 0.001) and 80 µM (P < 0.01). 

*Cytotoxicity of Aloctin-Imatinib combination against cancer cell lines*

Aloctin-IM combinations were assayed on AGS human gastric adenocarcinoma and Saos-2 human osteosarcoma which were the two most sensitive cells to Aloctin. The results of the Aloctin-IM combination treatment of AGS and Saos-2 cells are presented in Table 4.

**Figure 4:**
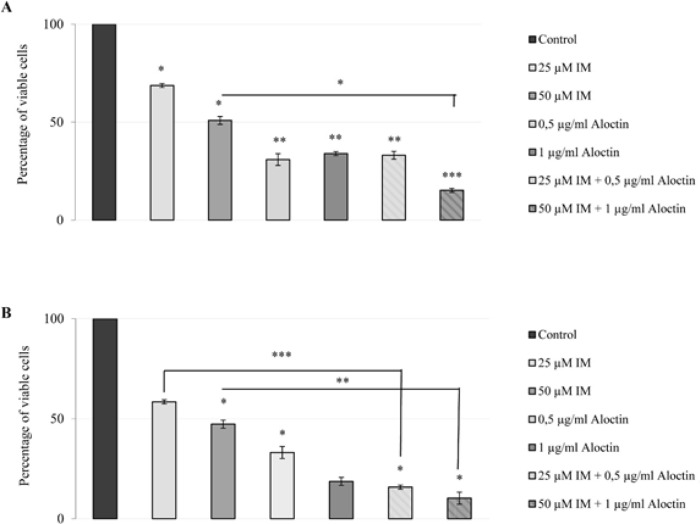
Cytotoxic Effect of Aloctin-IM Combination Treatment on A) AGS Human Gastric Adenocarcinoma Cells. B) Saos-2 human osteosarcoma cells. Cell viability (%) level measured by MTT assay after 72 hours treatment with different concentration of Imatinib (IM; 25 µM, 50 µM), Aloctin (0.5 µg/mL, 1 µg/mL) and combination of IM and Aloctin (25 µM + 0.5 µg/mL, 50 µM IM + 1 µg/mL). 25 µM IM + 0.5 μg/mL Aloctin concentration showed significant differences, compared to 25 µg/mL IM group in AGS cells. 0.5 µM IM + 1 μg/mL Aloctin concentration showed significant differences, compared to 50 µg/mL IM group in AGS and Saos-2 cells. *P < 0.05; **P < 0.01; ***P < 0.001

For AGS cells 1 µg/mL Aloctin combined with 50 µM IM, exerted significant difference (P < 0.05) in cytotoxic activity compared to the same dose of IM alone (Figure 4A). The results were more spectacular for Saos-2 cells, for which significant enhance in cytotoxicity compared to the same dose of IM alone was found for 0.5 µg/mL Aloctin combined with 25 µM IM (P < 0.001) as well as 1 µg/mL Aloctin combined with 50 µM IM (P < 0.01) (Figure 4B).

*Apoptosis/Necrosis analyses undertaken by flow cytometry*

**Figure 5:**
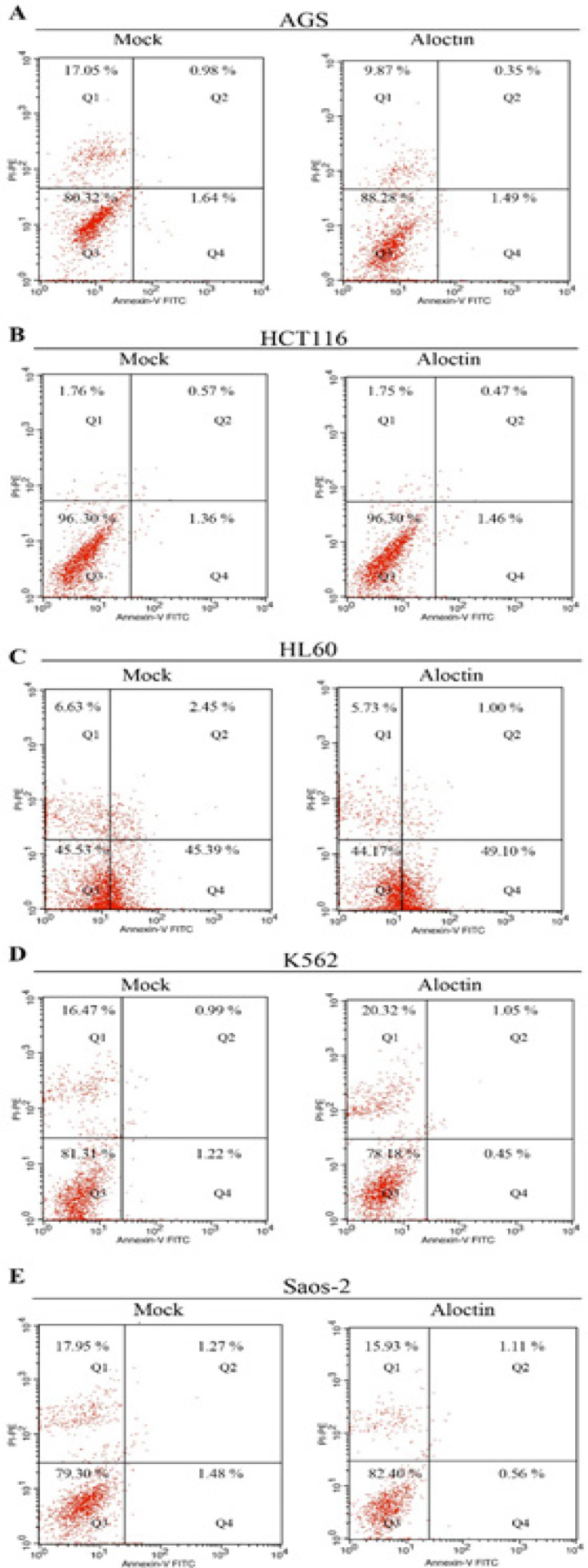
(A), AGS Cells Treated with the Dose of IC_50_ Aloctin (0.5 µg/mL) for 72 h . 2 × 10^4^ Cells were Analysed in each Sample and then Subjected to Annexin V/PI Staining and Flow Cytometric Analysis. Representative images of flow cytometry are shown. (B), Representative dot plots of HCT116 cells preincubated with culture media as negative control (Mock), HCT116 cells were treated with the dose of IC_50_ Aloctin (1.5 µg/mL) for 72 h and then subjected to Annexin V/PI staining and flow cytometric analysis. Representative images of flow cytometry are shown. (C), Representative dot plots of HL60 cells preincubated with culture media as negative control (Mock), HL60 cells were treated with the dose of IC_50_ Aloctin (1 µg/mL) for 72 h and then subjected to Annexin V/PI staining and flow cytometric analysis. Representative images of flow cytometry are shown. (D), Representative dot plots of K562 cells preincubated with culture media as negative control (Mock), K562 cells were treated with the dose of IC_50_ Aloctin (1 µg/mL) for 72 h and then subjected to Annexin V/PI staining and flow cytometric analysis. Representative images of flow cytometry are shown. (E), Representative dot plots of Saos-2 cells preincubated with culture media as negative control (Mock), Saos-2 cells were treated with the dose of IC_50_ Aloctin (0.5 µg/mL) for 72 h and then subjected to Annexin V/PI staining and flow cytometric analysis. Representative images of flow cytometry are shown. Q1: Necrosis, Q2: Late apoptosis, Q3: Alive, Q4: Early apoptosis

The results of Annexin V-FITC/PI assay apoptosis/necrosis analyses of the activity of Aloctin on cancer cells is are summarized in Figure 5. The results of flow cytometric studies on Aloctin treated cells revealed that Aloctin did not exert its cytototoxic activity by apoptosis or necrosis mechanisms.

**Table 1:** Purification Steps of Aloctin from *A. vera* Leaf Skin Extract (Starting from 103 g *A. vera* leaf skin)

Purification step	Volume (ml)	Protein (µg/ml)	Total protein (µg)	HA titer	HU/mg	Purification
Crude extract	200	41.30 ± 0.73	8260.3 ± 46.3	8	12.8	1
50 % amonium sulphate cut	25	162.87 ± 3.19	4071.86 ± 79.66	8	51.2	4
Affinity chromatography	7.9	151.69 ± 4.38	1198.31 ± 34.10	32	192.8	15.06

HA, hemagglutination; HU, hemagglutination units

**Table 2:** Results of Hemagglutination Inhibition Test Used to Determine Lectin Specific Carbohydrate Units

Carbohydrate Concentration (mM)																		
			200		100		50			25		12,5			6,25		PBS	
D-Maltose			Hemolysis		Hemolysis		Hemolysis			-		-			-		-	
D (+) Raffinose			-		-		-			-		-			-		-	
Melibiose			Hemolysis		Hemolysis		-			-		-			-		-	
D-Mannose			-		-		-			-		-			-		-	
N-Acetyl D-galactosamine			Hemolysis		Hemolysis		+/-			-		-			-		-	
Chitin			Hemolysis		Hemolysis		Hemolysis			-		-			-		-	
Glycoprotein		Concentration (mg/mL)																	
	10	5		2.5		1.25		0.625	0.31		0.156		0.078	0.039		0.0195		PBS	
Fetuin	+	+		+		+		+	+		+		+/-	+/-		+/-		-	
Avidin	+	+		+		+		+	+		+		+	+/-		-		-	
Mucin	Hemolysis	Hemolysis		Hemolysis		Hemolysis		+/-	+/-		+/-		+/-	+/-		-		-	
İnulin	-	-		-		-		-	-		-		-	-		-		-	

(+), There is hemagglutination inhibition; (-), No hemagglutination inhibition; (+/-), Poor hemagglutination inhibition.

**Table 3:** IC_50_ Values of Aloctin, AE and IM on Cells

Cell line	Aloctin (µg/mL)	AE (µM)	IM (µM)
AGS	< 0.3	19.03 ± 0.25	65 ± 1.31
HCT116	1.42 ± 0.07	> 150	50 ± 2.60
HEP3B	0.55 ± 0.02	201.64 ± 1.43	75 – 100
HL60	1.03 ± 0.07	20.93 ± 1.96	25 ± 0.51
K562	0.97 ± 0.06	60.98 ± 0.90	10 ± 0.34
Saos-2	<0.3	33.44 ± 0.68	108 ± 5.62

**Table 4:** Results of IM-Aloctin Combination Tests for AGS and Saos-2 Cells

Treatment dose	Viable cells (%)	
	AGS	Saos-2
25 µM IM	57.37*	68.72*
50 µM IM	47.28*	50.90*
0.5 µg/mL Aloctin	33.10**	30.89*
1 µg/mL Aloctin	18.65**	33.54
25 µM IM+0.5 µg/mL Aloctin	15.74**	33.11*
50 µM IM+1 µg/mL Aloctin	9.66 ***	15.20*

The tests were done in triplicate Student’s t test was applied in comparison to control untreated cells. * P < 0.05; **P < 0.01; ***P < 0.001.

## Discussion

As every lectin binds to specific carbohydrate residues or sugar moieties of glycoproteins situated on cell surfaces, it is important for research in lectin histochemistry, as well as for further lectin purification by affinity chromatography, to determine the specific ligand of every lectin. In our previous researches only N-acetyl galactosamine (Akev and Can, 1999) and ovalbumin (Ozsoy et al., 2012) were found to inhibit hemagglutination of Aloctin. Ovalbumin was thus used in affinity chromatography purification of Aloctin. In the present study, in a search for a new ligand, none of the different simple carbohydrates assayed has shown inhibition of hemagglutination. Among the new glycoproteins used, the fact that fetuin, avidin and musin have shown hemagglutination inhibition, tend us to propose these substances in further research as ligands for Aloctin.

In previous years lectins were presented as toxic substances, but nowadays this nocive property could be a chance for their use as antitumour agents. It was shown that lectins play role in apoptosis and autophagy (Liu et al., 2010;). As new strategies, especially based on plant derived chemotherapeutic agents have begun to emerge, research on the antitumour effect of lectins has gained importance. *Pisum sativum* and *Momordica charantia *seed lectins have been reported to exert in vivo cytotoxic activity on *Ehrlich ascites* tumours (Kabir et al., 2013). 

The first reports on the antitumour effect of *A. vera* lectin date from the 1980’s (Winters et al., 1981; Imanishi et al., 1981; Yagi et al., 1985). There are few recent reports on the in vivo antitumour activity of affinity chromatography-purified Aloctin (Akev et al., 2007b; Kaur et al., 2011). To date there is only one research undertaken on the in vitro cytotoxic effect of *A. vera* lectin in which HCT-15, HT-29 ve SW-620 colon cancer cells and HOP-62 lung cancer cells were used. However the mechanism of this cytotoxic effect was not elucidated (Kaur et al., 2011). To our knowledge this is the first comprehensive study, where different cancer cell lines were used for both Aloctin and AE and that apoptosis/necrosis mechanisms were investigated. AGS stomach and Saos-2 bone cancer cells were the most sensitive cells to Aloctin cytotoxicity. The fact that the cytotoxic effect was not due to apoptosis or necrosis urge the need for further investigation on the antitumour mechanism. In early studies, the mechanisms of the cytotoxic effects of *A. vera* lectin was related to the immunomodulatory activity (Imanishi et al., 1986; Yoshimoto et al., 1987; Winters, 1993).

Among the multiple substances found in *A. vera*, the anthraquinone glycoside AE is the most extensively studied one in regard to its antitumour effect. In several studies, AE has been reported to regress tumour growth (Lin et al., 2006; Chiu et al., 2009). Cui et al., (2008) found an IC_50_ value of 8,7µM for HCT116 cells treated with AE, contrarily to our study where HCT116 was the less affected cell line with an IC_50_ of > 150 µM. For K562 cells, Mahbub et al., (2013) gave an IC_50_ value of 500 µM, whereas in our study, this value was found 60.98 ± 0.90 µM. In the present study, AE showed the best cytotoxic effect on AGS (IC_50_19.03 ± 0.25 µM) and HL60 (IC_50 _20.93 ± 1.96 µM) cells. IM, which is a commonly used chemotherapeutic drug, in turn showed the best cytotoxic effect on K562 (IC_50_10 ± 0.34 µM) and HL60 (IC_50_ 25 ± 0.51 µM) cells. The cytotoxic effect of Aloctin revealed to be higher than IM on AGS and Saos-2 cells. Therefore in Aloctin-IM combination studies, which were undertaken for the first time, the cytotoxicity was significantly enhanced in both doses tested. 

We can suggest that Aloctin and aloe emodin alone or in combination are potential targets for anticancer drug research.
